# Supported employment: Meta-analysis and review of randomized controlled trials of individual placement and support

**DOI:** 10.1371/journal.pone.0212208

**Published:** 2019-02-20

**Authors:** Donald E. Frederick, Tyler J. VanderWeele

**Affiliations:** 1 Human Flourishing Program, The Institute for Quantitative Social Science, Harvard University, Cambridge, MA, United States of America; 2 Department of Epidemiology, Harvard T.H. Chan School of Public Health, Harvard University, Boston, MA, United States of America; Sapienza University fo Rome, ITALY

## Abstract

Supported employment is a treatment whereby those with severe mental illness (or other disabilities) receive aid searching for competitive employment and mental health (or other) treatments concurrently. The most popular implementation of supported employment is individual placement and support (IPS). We conducted meta-analytic analyses of the randomized controlled trials of IPS. We found that subjects in IPS, compared to usual treatment conditions, had better vocational outcomes (obtained any competitive employment: RR = 1.63, 95%CI = [1.46, 1.82]; job tenure: d = 0.55, 95%CI = [0.33, 0.79]; job length: d = 0.46, 95%CI = [0.35, 0.57]; income: d = 0.48, 95%CI = [0.36, 0.59]) Non-vocational outcomes estimates, while favoring IPS, included the null (quality of life: d = 0.30, 95%CI = [-0.07, 0.67]; global functioning: d = 0.09, 95%CI = [-0.09, 0.27]; mental health: d = 0.03, 95%CI = [-0.15, 0.21]). Analysis of the expected proportion of studies with a true effect on non-vocational outcomes with d>0.2 showed some reason to expect a possible improvement for quality of life for at least some settings (Prop = 0.57, 95%CI = [0.30, 0.84]).

## Introduction

In this article we present a series of meta-analyses of randomized controlled trials from the supported employment literature focused on the individual placement and support (IPS) treatment. We examine both vocational (*e*.*g*., competitive employment rates) and non-vocational (*e*.*g*., quality of life) outcomes. We hypothesized that IPS would lead to better vocational outcomes. We also hypothesized that IPS would lead to better non-vocational outcomes, as work may be a pathway to overall flourishing [1].

In our society, it is generally thought that working is better than not working. There is the obvious necessity for the vast majority of persons to work in order to sustain life in our current economic system. Beyond that, persons often report that they derive a great sense of meaning from their work. In his seminal book *Working*, Studs Terkel takes us into the lives of Americans and relates their stories and shows us just how much meaning people derive from and through their work [2].

Beyond narrative accounts, there is evidence that working may be associated with improved aspects of wellbeing such as mental health [3, 4]. How we go about and think about work (*e*.*g*., job crafting) may also impact the level of work engagement [5], which has been associated with increased levels of meaning [6]. Job crafting may also impact meaning via person-job fit [7].

There are also known associations between work, or more properly the lack of work, and negative outcomes. The most well-cited is the link between unemployment and suicide [8]. In that study, Nordt et al. estimated that one in five suicides might be attributable to unemployment for the period of 2000–2011 within sixty-three countries sampled [8].

All of this is by way of stating the obvious–work is a central part of most of our lives. Either directly or indirectly work appears to be significantly related to the modification of individual’s wellbeing. Unemployment may be associated with negative outcomes [9, 10], such as suicide, in the most extreme. Employment may be associated with positive outcomes such as increased physical and mental health [11–13]. These effects could arise because work is expected within our societies and therefore having and maintaining work is necessary for ‘fitting’ in to expected roles. It may also be an issue of time, activity, and relationships. We spend a large amount of lives in work and as such the workplace may provide the locus in which we experience our lives and a forum for developing relationships. Alternatively, it could be that there is something about work itself, something about the act of making and contributing, that is a mechanism that contributes to well-being [1].

Not all who wish to work can work or are able to find work. There are a substantial number of persons who are unemployed. The current unemployment rate in the United States stands at 4.0% as of June 2018 (https://data.bls.gov/timeseries/LNS14000000). Just nine years ago at the height of the recession unemployment reached its peak at 10% in October 2009 (https://data.bls.gov/timeseries/LNS14000000). These unemployment statistics include those who are looking for work, but who are not currently working. The statistics do not include those who have stopped looking for work and are assumed to be effectively out of the workforce. In 2014, the National Alliance on Mental Illness (NAMI) reported that unemployment levels for persons “receiving public mental health services” was at 80% nationally (https://www.nami.org/Press-Media/Press-Releases/2014/Mental-Illness-NAMI-Report-Deplores-80-Percent-Une). The NAMI report also stated “approximately 60% of the 7.1 million receiving public mental health services want to work”. We can see that there is a significant gap within this group between the number who say that they want to work and the number who do work. An obvious question then is how might we go about helping those who wish to find work do so.

One intervention that appears to be quite effective at helping those with SMI find work is the individual placement and support (IPS) treatment that is derived from supported employment (SE) theory. Past meta-analyses have estimated that supported employment increases the likelihood of achieving competitive employment by 2.4-fold (RR = 2.40 95%CI = [1.99, 2.90]) [14] and of finding any employment by 3.24-fold (RR = 3.24 95%CI = [2.17, 4.82]) [15]. What is more uncertain is to what degree does supported employment, in particular IPS, help decrease symptoms and provide for an overall better quality of life and wellbeing. We explore all of these issues in this article.

### What is supported employment?

Supported employment (SE) is a theory of work rehabilitation that argues that individuals–often called consumers–with some identified disability should be helped to find competitive employment (CE) at the beginning and not made to wait until after finishing some treatment or training program. Traditionally, the disability was severe mental illness (SMI), but has been expanded in recent years to cover a range of mental and physical conditions such as spinal cord injuries in veterans [16] as well as affective disorders [17]. SE may then be broadly construed so as to be part of the broader vocational rehabilitation literature (or perhaps even the broader vocational literature) and not just solely focused on SMI populations.

A bit confusingly, the term “supportive employment” is often used to refer to the type of treatment as well as the achievement of the main outcome, which is competitive employment [18]. Supported employment encompasses a fairly broad category that ought to be seen as a general theory of treatment and not a specific treatment in and of itself. Instead, the most popular implementation is individual placement and support (IPS). The essential point of SE and IPS is that treatment ought to offer subjects a concurrent means of receiving care and at the same time receive help in finding employment. This is in contrast to traditional programs, which we will often refer to as treatment as usual (TAU), in which subjects first receive treatment for their disability and then they would be helped to find employment. The difference can be succinctly stated: SE/IPS is ‘place-and-treat/train’ whereas TAU is ‘train/treat-then-place’.

Within the literature, authors enumerate different numbers of essential points to SE ranging from 6 to 8. Even the same authors may differ in the total number of essential points. For example, Bond lays out six key points in the form of headings in his paper [18]: “[1] Services focused on competitive employment… [2] Eligibility based upon choice; no exclusions … [3] Rapid job search … [4] Rehabilitation and mental health care integration … [5] Attention to patient's preferences … [6] Unlimited time and individual support”. In a later paper, the number of elements is listed as eight [3]: “[1] eligibility based on … choice, [2] focus on competitive employment …, [3] integration of mental health and employment services, [4] attention to client preferences, [5] work incentives planning, [6] rapid job search, [7] systematic job development, and [8] individualized job support ….”

The crucial point is not the exact enumeration of points, but as Bond [18] writes, it is the fact that SE casts aside three prior general assumptions. First that “people with SMI need an extended period of time in vocational preparation before entering a competitive job in order to become work ready and identify career goals", second that “rehabilitation services should be provided separately from mental health treatment services", and finally that programs should use transitional employment that "consists of time-limited job placements developed by a rehabilitation agency that [patients] work in preparation for competitive jobs."

To reiterate, the core of SE is the focus on individualized care oriented toward improving mental health and getting the subject employed (preferably in competitive employment) as soon as possible, which are done concurrently. Previous programs generally had a stepwise implementation wherein patients would receive clinical and pre-vocational training prior to seeking employment. Supported employment does away with this stepwise technique and replaces it with a concurrent approach.

Treatments based upon SE have been deployed in at least 38 of the 50 states in the United States [19]. Of those states, Johnson-Kwochka et al. report that there was an average of 13.7 programs per state (SD = 16) and there were a total of 523 programs [19]. There are a number of SE implementations that have been designed and tested internationally (See Results; See [14] for a meta-analysis focused on this topic). Clearly, the use of SE is not limited to a few centers or isolated to a single country. There are also many resources for finding how to do SE/IPS available online. For example, the IPS Employment Center (https://www.ipsworks.org/), which also provides online training courses (https://www.ipsworks.org/training-consultation-services/), has a large collection of published books and manuals (https://www.ipsworks.org/order/books/.

In this present work, we searched the literature for randomized controlled trials of supported employment that employed individual placement and support. We then performed random effects meta-analyses on a number of outcomes, including the most widely used one–competitive employment. We also used a new statistical measure to estimate the proportion of settings in which we would expect to find a scientifically meaningful effect [20].

## Methods

### Literature search and data extraction methods

Our search goal was to identify the extent literature of randomized controlled trials that used individual placement and support to test supported employment against treatment as usual controls. To this end, we searched PSYCHinfo for articles. Exact terms and results are detailed below in the Results section. Relevant data were extracted from the articles and entered into a PostgreSQL database (v.9.6). The first author did the search and data extraction. We have included the PLoS One checklist as supporting information (S1 File).

### Statistical methods

We performed random effects meta-analyses in R (v.3.2.1) using the metafor package [21]. Data from the PostgresSQL database were queried and imported into R using r-postgresql library for analysis. For reproducibility, we saved the relevant data as R data files and have included them as supplemental materials along with the needed R script file (S2 and S3 Files, respectively).

For binary outcomes (*e*.*g*., subjects attaining competitive employment) we computed the log relative risk. We convert from log(RR) to RR when needed for comparisons to previous research. For continuous data (*e*.*g*., number of weeks worked), we computed the standardized mean difference from the given means and standard deviations. In some cases standard deviations were not directly provided in the articles. In these cases, we computed the needed standard deviations via established methods (*e*.*g*., estimating standard deviations standard errors or confidence intervals).

In addition to the random effects meta-analyses, we also estimated the proportion of scientific meaningful effect sizes across various settings examined in the meta-analysis [20]. For risk ratios we set the scientific meaningful values at 1.2 and 0.8. For standardized mean differences we set the scientific values of interest at 0.2 and -0.2. We performed these analyses in R using the EValue library on samples that had five or more articles. Such proportion metrics can be especially helpful when there is substantial heterogeneity in effect sizes across studies and settings. For example, a pooled estimate close to the null may either be due to most of the effect sizes being close to null, or because several are large and positive while others are large and negative. The proportion metrics help to distinguish these scenarios.

## Results

### Literature search results and data extraction

**Fig 1:**
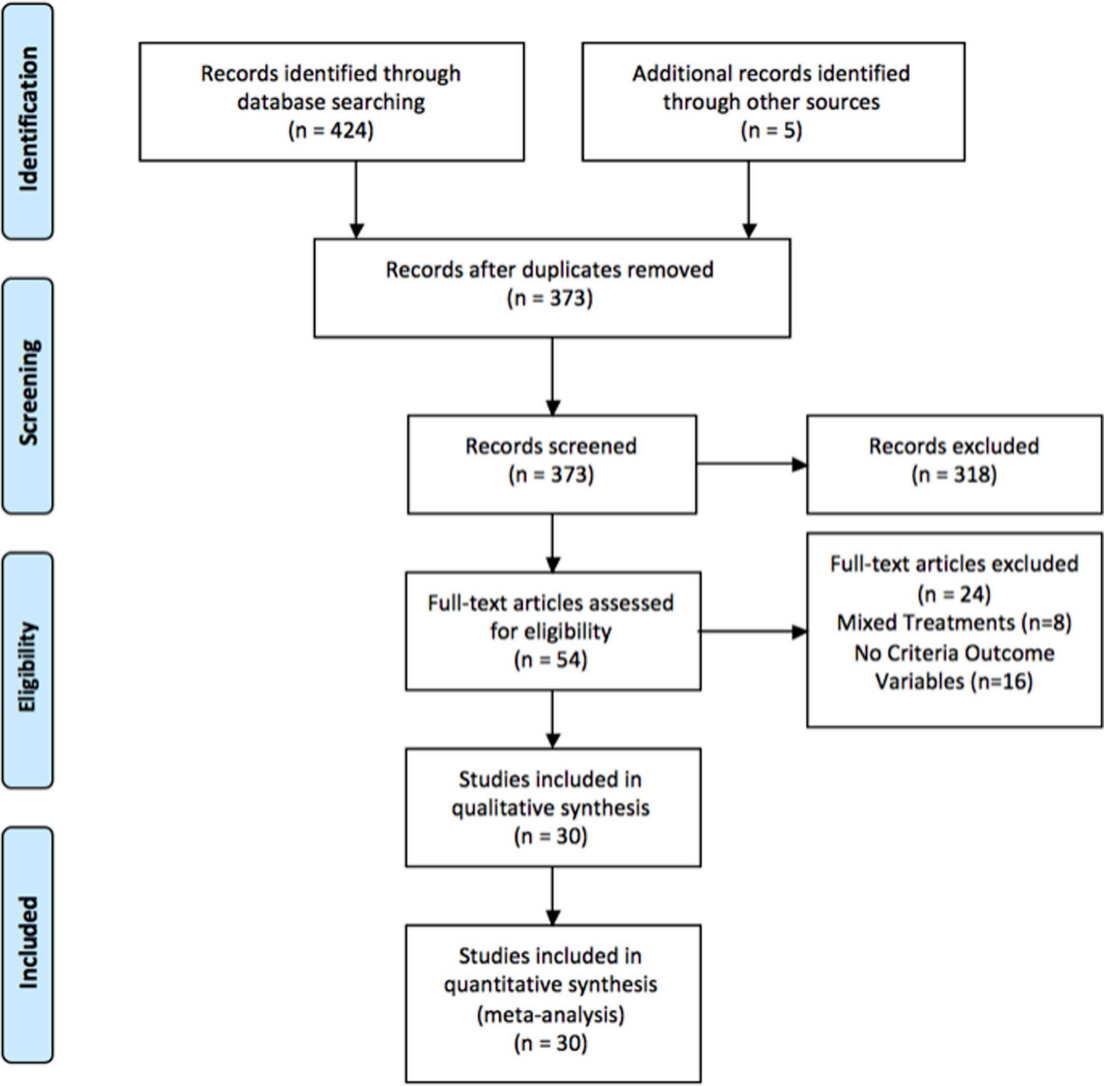
PRISMA flow chart. Our literature search found 368 unique articles and we found another 5 that were referenced in articles. We initially screened articles to check that they used an RCT design, extended the duration of a previous RCT experiment, or performed secondary analysis on a previous RCT. We found that 49 articles met this criterion plus the 5 that we found referenced in other articles. We then read those articles and excluded ones that used a mixed treatment (*e*.*g*., IPS plus some other component) or did not report an outcome variable of interest. We excluded 24 articles for these reasons. This left 30 articles in total that we used for analysis.

Search was conducted using PSYCHInfo database by the first author (See Fig 1 for PRISMA flowchart and Table 1 for queries). The primary query was “supported employment AND randomized controlled trial”, which returned 111 results. A larger search with “supported employment” with methodology filters (clinical trial, follow-up study, treatment outcome, prospective study, clinical case study, focus group, retrospective study, and nonclinical case study) was also done in order to find any studies that may have been misclassified or had not contained the keywords “randomized controlled trial”. This larger search returned 313 results. After de-duplication, we were left with 368 publications.

**Table 1:** Search terms used and number of results.

Search Query	Methodology Filters	#Results
supported employment AND randomized controlled trial	n/a	111
supported employment	clinical trial, follow-up study, treatment outcome, prospective study, clinical case study, focus group, retrospective study, and nonclinical case study	313

In order to retain an article it had to have performed or done analysis on a RCT of IPS vs. a treatment as usual control. The outcome variables had to have been either the usual work outcomes (*e*.*g*., competitive employment achieved, about of time in work) and/or non-work outcomes (*e*.*g*., quality of life).

After deduplication, we screened the articles for methodology by reading abstracts, keywords, and subject headers. We retained only articles that appeared to have done RCT trials, did a follow-up to or extension of a previous RCT (*i*.*e*., included additional follow-up time), or performed secondary analysis on a previous RCT. We did not retain articles that were cross-sectional, prospective, retrospective, or case studies. We did not retain articles that reported results from just a single arm of RCT studies. We did not retain studies that compared IPS to a better/different version of SE unless there was a third control arm of the study that was treatment as usual. We did retain articles that appeared to be secondary analyses on previous RCT studies in order to see if new outcomes of interest were reported. After screening we were left with 49 articles.

We then read the papers to ensure that they had the needed outcome measures and were in fact RCT studies between IPS and a treatment as usual control group. We removed 26 articles. Of those, we removed studies that used IPS plus some other treatment (n = 8). The remaining articles were removed because there was not the required outcome variable present.

While reading the articles, we noted any references to other RCT studies that used IPS as a treatment. We made sure that any RCT studies that were referenced were cross-referenced to our list. If the study was missing, we sought it out and checked it. If it met our inclusion criteria, we retained the study. We found seven additional articles by this method of which four met our criteria [16, 22–24]. Finally, we also found one additional article [25] from our reading of a previous meta-analyses [26].

In total, we found 30 articles [16, 22–25, 27–50] for our meta-analysis that matched our criteria (Tables 1–3 and Fig 1). Of those, 25 were original RCTs (Table 2), two were follow-ups that extended the period of observation of a previous RCT (Table 3), and three were secondary analyses on a previous RCT (Table 4). All articles reported the results of randomized controlled trials (RCT) that focused on comparing supported employment (SE) as implemented via individual placement and support (IPS) to a control condition, which was usually the locally available best usual practice.

**Table 2:** Studies found.

Article	Duration (Months)	Control	N Randomized	
			IPS	TAU
Bejerholm et al. 2015	18	TVR	60	60
Bond et al. 2007	24	Diversified placement approach (DPA)	96	98
Bond et al. 2015	12	Work Choice (A Job Club Approach)	43	44
Burns et al. 2007^a^	18	Vocational Services	156	156
Davis et al. 2012	12	Veterans Health Administration Vocational Rehabilitation Program (VRP)	42	43
Drake et al. 1996	18	Group Skills Training	74	69
Drake et al. 1999	18	Enhanced vocational rehabilitation (EVR)	76	76
Drake et al. 2013	24	TAU	1121	1117
Heslin et al. 2011	24		109	110
Hoffmann et al. 2012	24	TVR	46	54
Howard et al. 2010	12	TAU	109	110
Latimer et al. 2006	12	TAU	75	75
Lehman et al. 2002	24	TAU	113	106
Li-Tsang et al. 2008	1	Self Placement	32	31
Michon et al. 2014 ^a^	30	TVR	71	80
Mueser et al. 2004	24	TAU	68	69
Oshima et al. 2014	6	TAU	18	19
Ottomanelli et al. 2012	12	TAU	81	76
Poremski et al. 2017	8	TAU	45	45
Tsang et al. 2009 ^a^	15	TVR	56	55
Twamley et al. 2012	12	CVR	28	22
Viering et al., 2015	24	TAU	127	123
Waghorn et al. 2014	12	TAU	106	74^a^
Wong et al. 2008	18	TAU	46	46
Zhang et al. 2017	15	TAU	54	54

a. 28 of the original TAU subjects switched to the intervention at 6months. We have subtracted the 28 from the original randomized count for our analysis.

**Table 3:** Follow-up studies.

Article	Original Duration (Months)	Total Duration (Months)	Follow-up To What Study
Hoffman et al. 2014	24	60	Hoffman et al. 2012
Ottomanelli et al. 2014	12	24	Ottomanelli et al. 2013

**Table 4:** Secondary analyses of existing studies.

Article	Original Article	New Data
Burns et al. 2009	Burns et al. 2007	quality of life, global functioning, mental health
Kukla et al. 2013	Bond et al. 2007	quality of life, mental health,
Ottomanelli et al. 2013	Ottomanelli et al. 2012	quality of life

In general, research articles varied greatly in quality, as has been previously reported [15]. Researchers measured the same outcome variable in a number of ways. For example, competitive employment was often measured as having had any competitive employment during the study period and/or as having been employed at the end of the study.

A number of researchers did calculate the number of needed subjects based on expected effect sizes and attrition rates. However, the effect size estimates used were generally for the main outcome, which was usually competitive employment. This effect (as shown below) is much larger than other possible effects (*e*.*g*., quality of life improvement). Because power was calculated only for the main outcome variable, researchers may have lacked sufficient power to detect secondary outcomes of interest. This provides a strong motivation for meta-analysis. We return to this point in the discussion.

Articles also varied by whether or not they reported measures of fidelity to the study protocol. A number of fidelity scales have been developed that include a shorter 15-question [51] and longer 25-question survey [52].

We performed an additional search for meta-analyses and found nine previous meta-analyses (query: “supported employment” with methodology filter ‘meta analysis’ in PsycINFO). We briefly detail some results from these previous articles. One meta-analysis used 14 studies and found significant effects on competitive employment over controls [15]. These authors note that in general the level of studies were low to very low quality. Still, the authors concluded that IPS increased levels of any employment (k = 7, RR = 3.24, 95%CI = [2.17, 4.82]), length of competitive employment (k = 1, MD = 70.63, 95%CI = [43.22, 94.04]), length of days in any employment (k = 2, MD = 9.86, 95%CI = [5.36, 14.36]), length of job tenure (k = 1, MD = 9.86, 95%CI = [5.36, 14.36]), increase in paid employment (k = 2, MD = 84.94, 95%CI = [51.99, 117.89]), and decreased time to first employment (k = 1, MD = -161.60, 95%CI = [-225.73, -97.47]). However, this study did not look at non-vocational outcomes.

A more recent meta-analysis looked at the possible moderation by country of treatment [14]. The authors found that there did not appear to be substantial variation by country and that IPS consistently outperformed controls in helping subjects find competitive employment (RR = 2.40, 95%CI = [1.99, 2.90]).

A third meta-analysis looked at supported employment effects on non-vocational outcomes [53]. The researchers found a significant effect on quality of life with those in IPS having a higher level (d = 0.28, 95%CI = [0.04, 0.52]), but no significant effects on global functioning (d = -0.01, 95%CI = [-0.13, -0.11]) or mental health symptoms (d = -0.20, 95%CI = [-0.62, 0.23]).

Although there have been previous meta-analyses, our current paper contributes in ways beyond prior meta-analyses in including more studies, a broader range of outcomes, examining length of experiment as a moderator, and using a novel statistic technique to estimate the proportion of settings with scientific meaningful effect sizes, which is able to help interpretation of meta-analytic researches with significant heterogeneity.

### Competitive employment status

Researchers investigated competitive employment outcomes in one of two general ways (Table 5). First, some researchers investigated if subjects obtained any competitive employment at any time during the study period. Second, other researchers looked at employment at the end of follow-up or repeatedly at follow-up time points.

**Table 5:** Competitive employment counts and percentages.

Article	Any				End			
	IPS		TAU		IPS		TAU	
	N	%	N	%	N	%	N	%
Bejerholm et al. 2015	-	-	-	-	19	32	5	8
Bond et al. 2007	69	72	32	33	29	30	15	15
Bond et al. 2015	13	30	3	7	-	-	-	-
Burns et al. 2007	85	54	43	28	-	-	-	-
Davis et al. 2012	32	76	12	28	-	-	-	-
Drake et al. 1996	526	47	347	31	281	25	158	14
Drake et al. 1999	57	77	27	39	30	41	15	22
Drake et al. 2013	45	59	7	9	30	39	14	18
Heslin et al. 2011	21	19	11	10	-	-	-	-
Hoffmann et al. 2014^a^	30	65	18	33	20	43	9	17
Howard et al. 2010	14	13	8	7	-	-	-	-
Killackey et al. 2008	32	70	13	28	20	43	10	22
Latimer et al. 2006	31	27	7	7	10	9	2	2
Lehman et al. 2002	27	84	19	61	-	-	-	-
Michon et al. 2014	31	44	20	25	-	-	-	-
Mueser et al. 2004	51	75	19	28	25	37	5	7
Oshima et al. 2014	8	44	2	11	-	-	-	-
Ottomanelli et al. 2014^b^	25	31	8	11	-	-	-	-
Poremski et al. 2017	23	51	18	40	-	-	-	-
Tsang et al. 2009	-	-	-	-	30	54	4	7
Twamley et al. 2012	17	61	8	27	9	32	4	13
Viering et al., 2015	63	50	41	33	51	40	34	28
Waghorn et al. 2014	45	42	13	18	-	-	-	-
Wong et al. et al. 2008	32	70	13	28	20	43	10	26
Zhang et al. 2017	27	50	18	33	-	-	-	-

a. Follow-up study to Hoffman 2012 that extended period of observation

b. Follow-up study to Ottomanelli 2012 that extended period of observation

We performed two different analyses. First, we looked at the most inclusive definition that counted an individual as competitively employed if they had been so at any point during the study. Second, we looked at the outcomes at only the end of the studies. When there was a follow-up study that added additional years of observation, we used that for analysis instead of the original study. Finally, given that we had access to the total number of subjects randomized, we performed the analyses following the intent-to-treat paradigm. In other words, for the Ns needed to be specified we used the total number of subjects randomized and not the values that the authors may themselves have used in their analyses.

**Fig 2:**
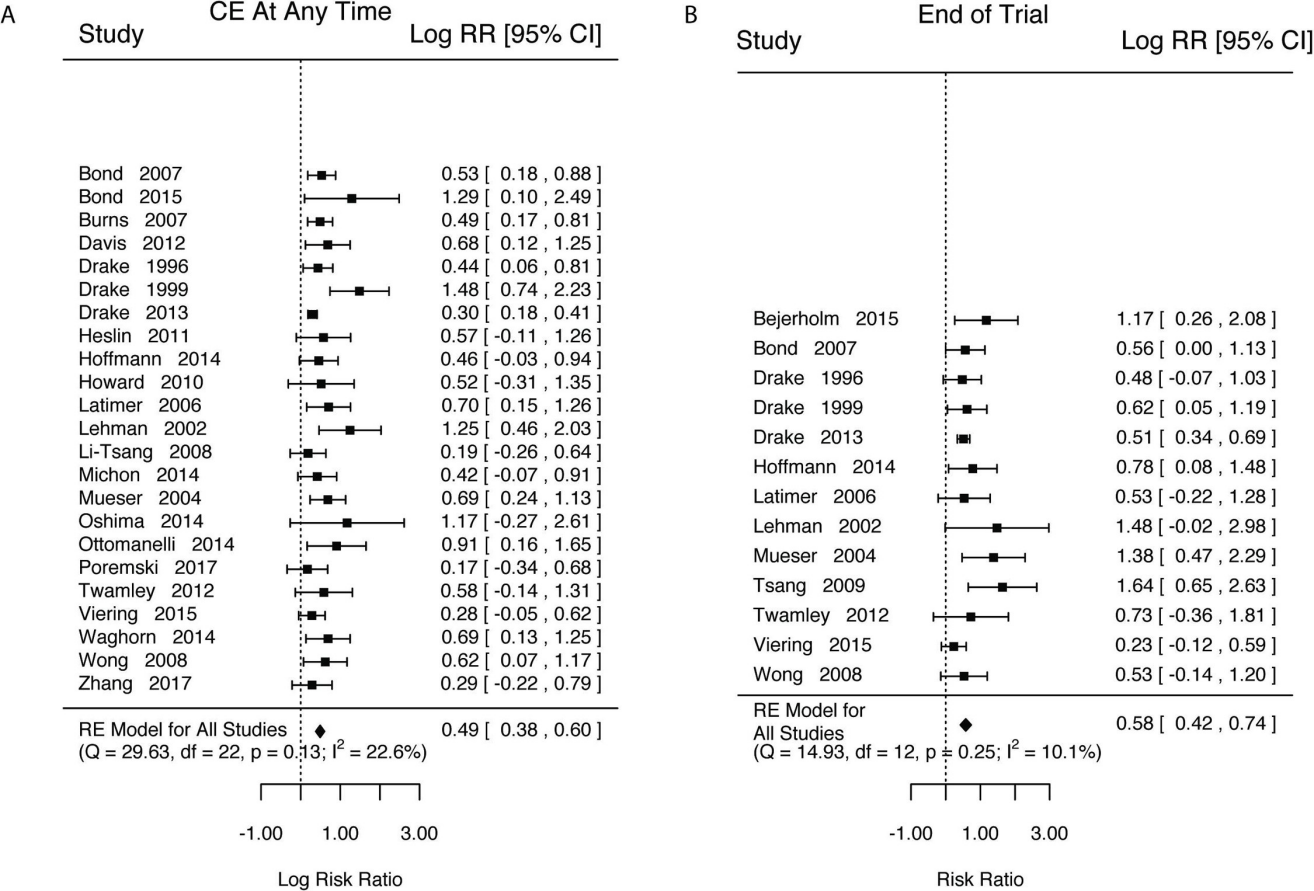
Competitive employment outcomes. A) For having held a competitive employment job for any portion of the experimental period B) For having held a competitive employment job at end of the experiment.

We estimated that those in IPS conditions were 1.6x more likely to have found CE at any point in the study compared with those in control conditions (Fig 2A, k = 23, log(RR) = 0.49, SE = 0.06, Z = 8.70, p<0.0001, 95%CI = [0.38, 0.60], τ^2^(SE) = 0.01(0.02), I^2^ = 22.59%, H^2^ = 1.29, Q(22) = 29.64, p = 0.1277). We also estimated that those in IPS conditions, compared to those in control conditions, were 1.8x more likely to have been competitively employed at the end of studies (Fig 2B, k = 13, log(RR) = 0.58, SE = 0.08, Z = 7.23, p<0.0001, 95%CI = [0.42, 0.74], τ^2^ (SE) = 0.01(0.03), I^2^ = 10.10%, H^2^ = 1.11, Q(12) = 14.93, p = 0.2451).

Given the variety of study lengths (Tables 2 and 3), we reran the above two analyses including study length as a moderator. Estimates for length as a moderator for finding CE at any point during the study or having been in CE at the end of the study both were close to zero and included the null (Any: Length = -0.0001, 95%CI = [-0.01, 0.01]; End: Length = 0.0005, 95%CI = [-0.02, 0.02]).

Our estimates showed that there was significant heterogeneity in the underlying study populations. In order to aid in interpretation we estimated the proportion of settings that the effects would exceed a scientifically meaningful threshold of RR = 1.2 or below the threshold of RR = 0.8. For finding CE during any part of a study, the proportion of studies with true RR>1.2 is estimated to be >0.99 (95%CI = [0.95, 1]), with the proportion of studies with true RR<0.8 expected to be <0.001 (95%CI = [0, <0.01]). Looking at CE at the end of follow-up, our estimates were very similar (RR = >1.2, Proportion>0.999, 95%CI = [>0.99, 1]; RR<0.8, Proportion = <0.001, 95%CI = [0, <0.01]).

### Secondary vocational outcomes

**Fig 3:**
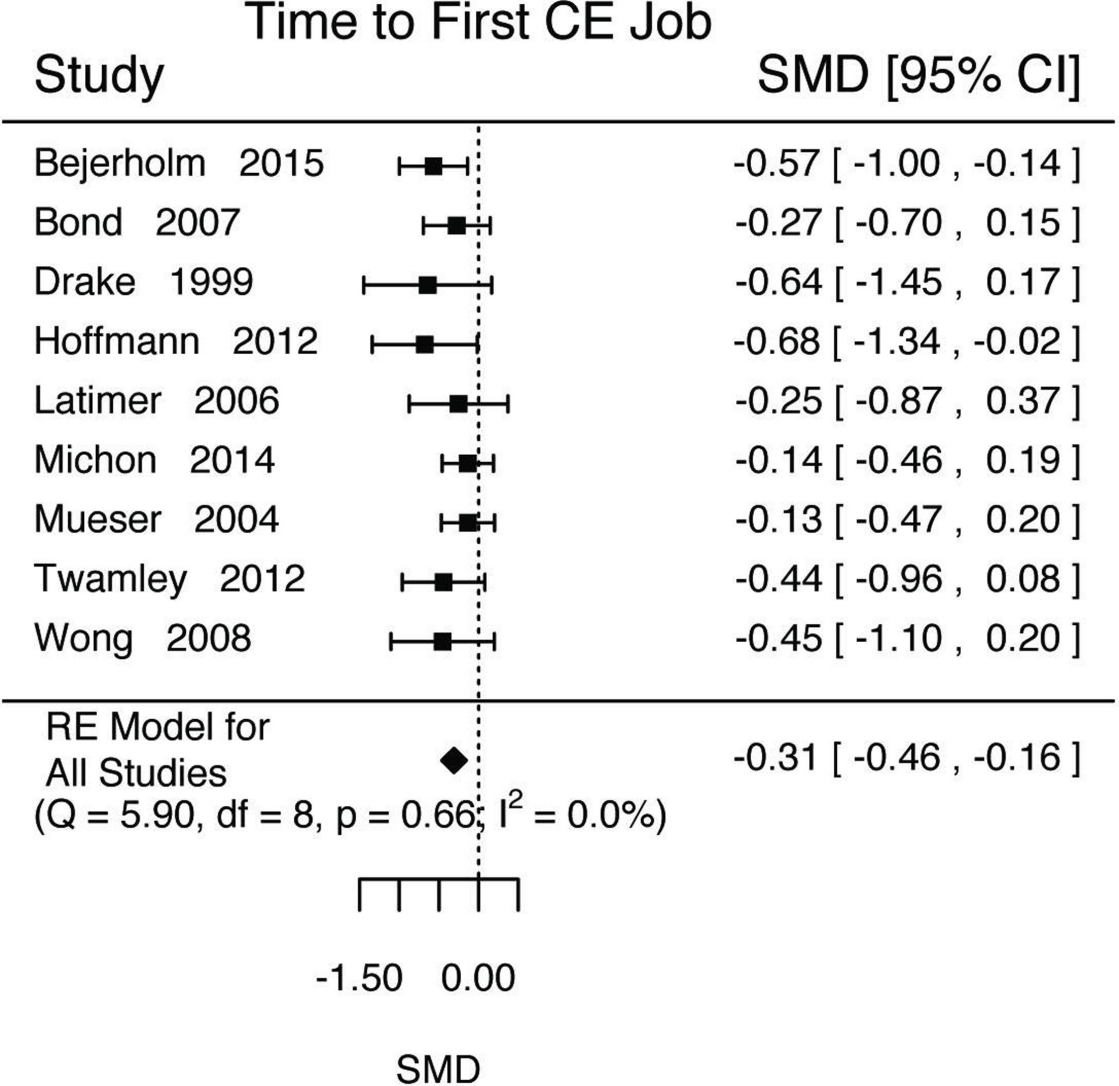
Time to first competitive employment job results.

**Fig 4:**
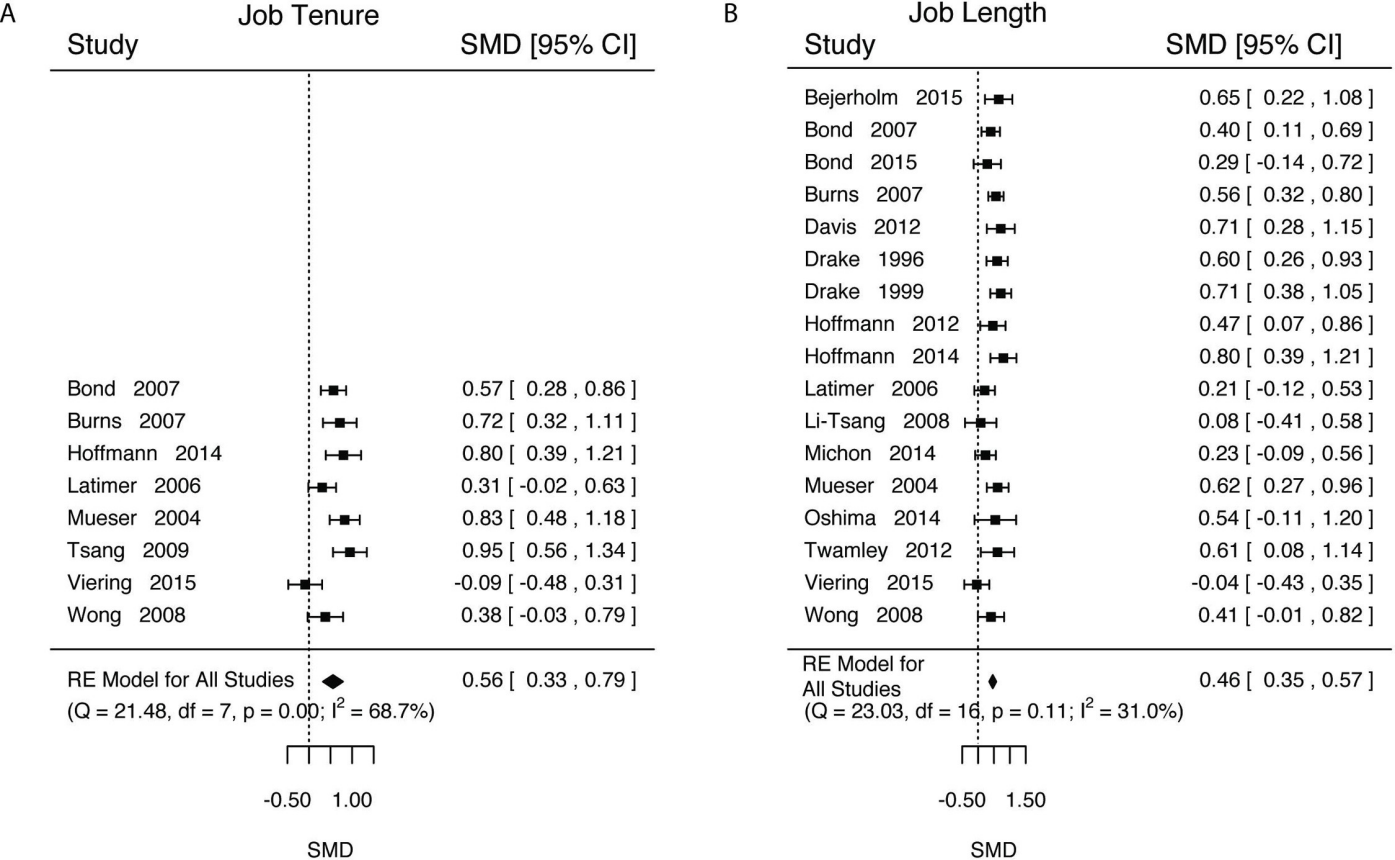
Competitive employment job tenure and job duration results. A) Job tenure, which is the duration of the longest held competitive employment job. B) Job duration, which is the total length of competitive employment.

**Fig 5:**
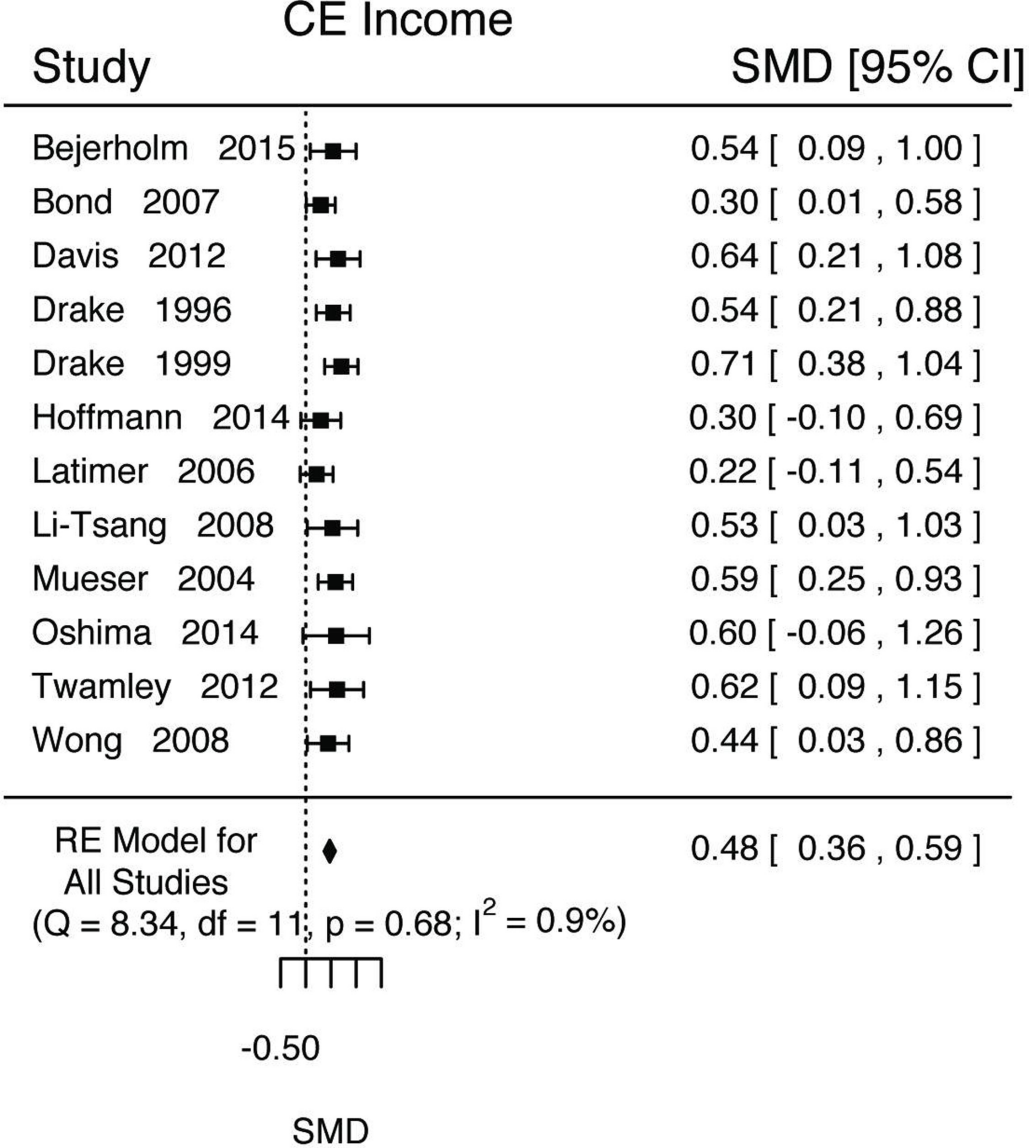
Income outcome results.

In addition to the main outcome of interest—competitive employment status—studies often measured and reported secondary vocational outcomes (Tables 6–10 and Figs 3–5). We performed meta-analyses on four of these (time to first competitive employment job, job tenure, total time worked, and income). We only performed meta-analysis from papers that only used competitive employment for these statistics. We omit from analysis statistics that combined all types of employment (*i*.*e*., competitive and not).

**Table 6:** Time (in days) to first competitive employment job.

Article	IPS			TAU		
	Mean	SD	N	Mean	SD	N
Bejerholm 2015	462.5	178	41	541.2	86	46
Bond 2007	156.41	122.26	69	193.44	156.5	32
Drake 1999	125.6	142.80	45	293.4	636.40	7
Hoffman 2012	116.7	155.5	27	214.3	106.5	14
Latimer 2006	126.3	95.6	35	152.9	123.3	14
Michon 2014	269.6	217.6	69	301.9	253.7	79
Mueser 2004	196.63	188.55	68	218.84	137.6	69
Twamley 2012	94.06	87.99	30	138	108.1	28
Wong 2008	72	77	32	118	143	13

**Table 7:** Competitive employment job tenure.

Article	IPS			TAU		
	Mean	SD	N	Mean	SD	N
Bond 2007 ^a^	27.51	33.11	92	11.06	23.94	95
Burns 2007 ^b^	213.6	159.42	83	108.4	111.95	39
Hoffman 2014^a^^,^^c^	104.8	102.7	46	35.5	68.8	54
Latimer 2006 ^a^	5.8	10.9	75	2.8	8.4	74
Mueser 2004 ^a^	25.54	31.05	68	5.3	14.89	69
Tsang 2009 ^a^	12.34	16.02	56	1.05	4.5	55
Viering 2015 ^b^	51.25	70.63	63	57.85	81.31	41
Wong 2008 ^b^	133	160	46	75	143	46

a. Weeks

b. Days

c. Hoffman 2014 instead of Hoffman 2012 was used.

**Table 8:** Competitive employment job length.

Article	IPS			TAU		
	Mean	SD	N	Mean	SD	N
Bejerholm 2015	196	384	41	19	82	46
Bond 2015	40.5	99.2	42	15.9	65.7	43
Bond 2007	596.28	836.02	91	284.61	722.95	95
Burns 2007	428.8	706.77	143	119.1	311.94	138
Davis 2012	656	661	42	236	494	43
Drake 1999	322	569.4739	74	27.6	124.6645	76
Drake 1996	607.03	842.59	74	205.13	400.09	69
Hoffman 2012	628	694.6	46	316.9	632.9	54
Hoffman 2014	106.8	103	46	37	70.1	54
Latimer 2006	126.4	266.8	75	72.5	251.6	74
Li-Tsang 2008	33.9	20.6	32	32.2	20.2	31
Michon 2014	422.2	920.1	69	236.8	648.1	79
Mueser 2004	372.57	515.6	68	102.84	338.27	69
Oshima 2014	167.7	300.3	18	40.6	129.9	19
Twamley 2012	10.5	13.5	30	3.61	7.8	28
Viering 2015	47.37	30.33	63	48.6	30.99	41
Wong 2008	136	161	46	74	141	46

**Table 9:** Income from competitive employment.

Article	IPS			TAU			Units
	Mean	SD	N	Mean	SD	N	
Bejerholm 2015	1294	545	34	1004	516	44	1294
Bond 2007	5034	8534	92	2675	7273	95	5034
Davis 2012	9264	13294	42	2601	6009	43	9264
Drake 1999	1875.47	3375.811	74	153.99	721.8211	76	1875.47
Drake 1996	3394.01	5446.25	73	1077.82	2237.84	67	3394.01
Hoffman 2014	11826	16917	46	6885	16102	54	11826
Latimer 2006	961.7	2162	73	520.8	1901	73	961.7
Li-Tsang 2008	4468	3145	32	2958	2434	31	4468
Mueser 2004	2078.41	2891.36	68	617.59	1943.25	69	2078.41
Oshima 2014	211.5	388.7	18	36.5	116.9	19	211.5
Twamley 2012	1857	2969	30	456	883	28	1857
Wong 2008	2300	3100	46	1100	2200	46	2300

**Table 10:** Quality of life measures.

Article	IPS			TAU			Scale
	Mean	SD	N	Mean	SD	N	
Burns 2009	4.7	0.758	132	4.7	0.861	120	Lancashire Quality of Life Profile
Drake 2013	4.23	1.47	1004	4.01	1.57	1051	Quality of Life Interview
Drake 1999	5	1.46	74	4.8	1.57	76	Quality of Life Interview
Heslin 2011	4.1	0.9	93	3.9	1.1	95	MANSA
Hoffman 2014	6.9	1.3	46	6.2	2	54	Wisconsin Quality of Life
Howard 2010	4.03	0.82	98	3.95	0.87	99	MANSA
Kukla 2013	4.7	1.55	92	4.71	1.47	95	Quality of Life Interview
Ottomanelli 2013	55.1	14.3	71	55	14.2	76	VC36-Mental Health
Zhang 2017	39.33	7.87	54	25.46	6.53	54	Personal Wellbeing Index

There were generally two methods in which estimates of the treatment for secondary vocational outcomes were derived. First, researchers estimated the effect using only those subjects that found a competitive employment job. Second, researchers used an intent-to-treat method whereby they imputed data with zeros of those that did not achieve competitive employment status. We include the estimates as given by the authors, which is a mix of the above two methods. If a study reported both intent-to-treat and conditional statistics, we selected to use the intent-to-treat values.

### Time to first competitive employment

Time to first competitive employment job was reported by seven studies (Table 6). We estimated that those in IPS would have a shorter time to their first competitive employment job (Fig 3, k = 9; d = -0.31, SE = 0.08, Z = -3.93, p<0.0001, 95%CI = [-0.46, -0.15], τ^2^ (SE) = 0(0.03), I^2^ = 0.00%, H^2^ = 1.00, Q(8) = 5.8994, p = 0.6585). Because there was not significant heterogeneity, we were unable to estimate a proportion. Experiment length was not a significant moderator (Length = 0.02, SE = 0.01, Z = 1.42, p = 0.16, 95%CI = [-0.01, 0.05]).

### Job tenure & length of competitive employment

The amount of time that a subject spent in or held a competitive employment job was measured in two ways. First, researchers reported job tenure or length of the longest held CE job per subject (Table 7). Second, researchers reported total time worked as the sum of all time in all CE jobs per subject (Table 8). Researchers reported total time in different units (e.g., hours, weeks). For our analysis, we selected to use the smallest unit that a researcher reported (i.e., we include hours if researchers reported both hours and weeks).

For job tenure, we estimated that persons in IPS held a CE job longer than those in control conditions (Fig 4A, k = 5, d = 0.56, SE = 0.12, Z = 4.72, p<0.0001, 95%CI = [0.33, 0.79], τ^2^ (SE) = 0.08(0.06), I^2^ = 68.68%, H^2^ = 3.19, Q(7) = 21.4770, p = 0.0031). Running the analysis with experiment duration as a moderator was not significant (Length: d = 0.005, 95%CI = [-0.01, 0.02]). We estimated that scientific meaningful effects that we expect to be observed with d>0.2 was 0.90 (95%CI = [0.68, 1]) and that the proportion of studies with d<-0.2 to be tiny with a confidence interval that included the null (Prop = 0.003, 95% = [0, 0.02]).

For total time worked in CE, we estimated that those in the IPS condition worked significantly more than controls (Fig 4B, k = 17, d = 0.46, SE = 0.06, Z = 8.23, p<0.0001, 95%CI = [0.35, 0.57], τ^2^ (SE) = 0.02(0.02), I^2^ = 31.03%, H^2^ = 1.45). Experiment length did not appear to be a significant moderator (Length: d = 0.005, 95%CI = [-0.004, 0.015]). We estimated the proportion of effect sizes of d>0.2 as 0.98, (95%CI = [0.86, 1]) and the proportion with d<-0.2 to be essentially zero (Prop = <0.001, 95%CI = [0, <0.001]).

### Income from competitive employment

In addition to the question of if a subject found a CE job and how much time they spent in the job, researchers also measured income (Table 9). For the following analyses, as above, we used only values that were derived from competitive employment (*i*.*e*., if the reported statistic was from income of competitive and non-competitive employment sources, we did not use it).

We estimated that those in IPS conditions would have significantly more income (Fig 5, k = 12; d = 0.48, SE = 0.06, Z = 8.31, p<0.0001, 95%CI = [0.36, 0.59], τ^2^ (SE) = 0.0004(0.02), I^2^ = 0.90%, H^2^ = 1.01, Q(11) = 8.3404, p = 0.6825). We estimated that we would expect all settings to have an effect size d>0.2 (Prop = 1, 95%CI = [1, 1]) and none to be in the opposite direction with d<-0.2 (Prop = <0.001, 95%CI = [0, <0.001]). Length was not a significant moderator (Length = -0.004, 95%CI = [-0.01, 0.005]).

### Non-vocational outcomes

In addition to the vocational outcomes reported above, we performed analyses on three non-vocational outcomes (quality of life, global functioning, and mental health).

### Quality of life

**Fig 6:**
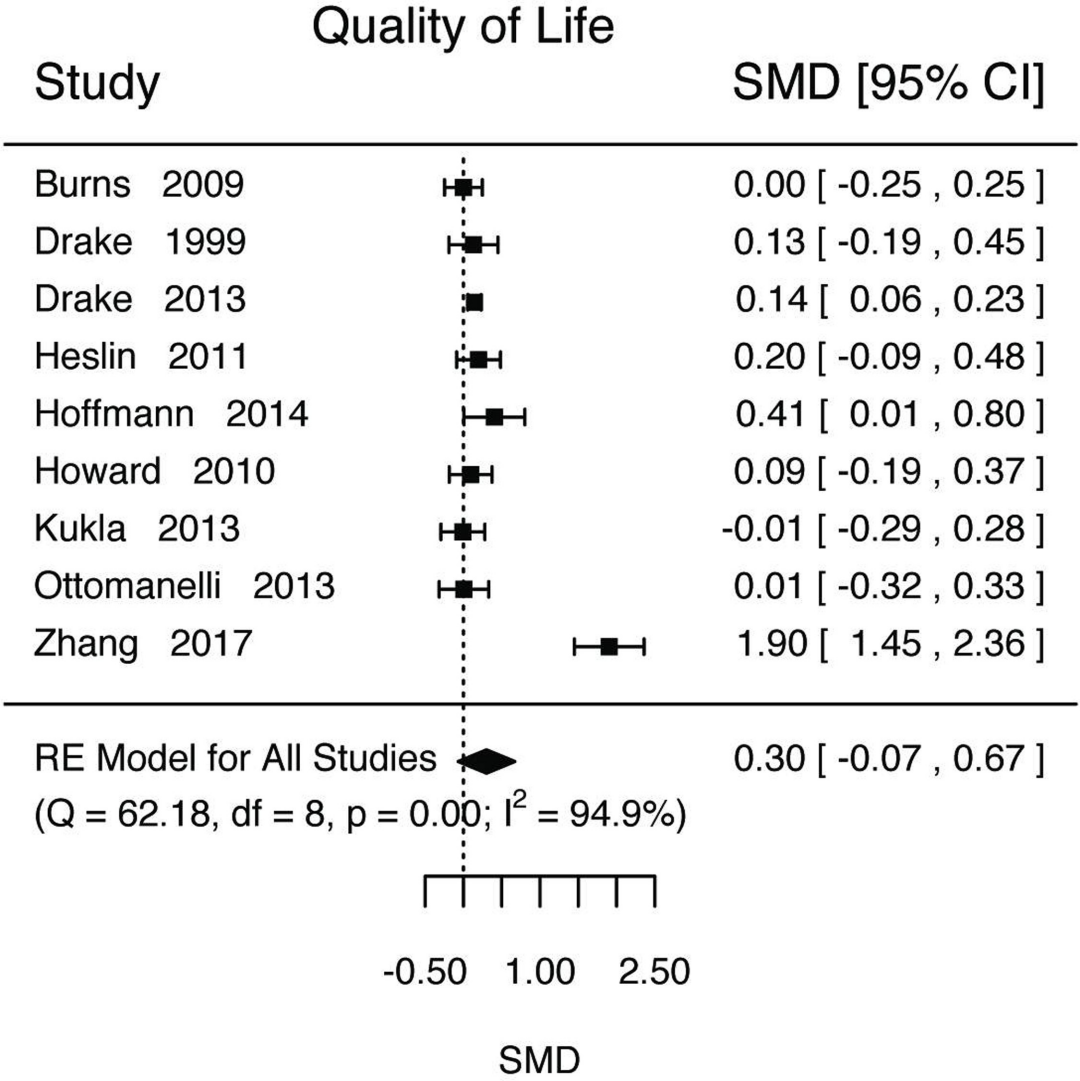
Quality of life outcome.

Researchers used various measures for quality of life, such as Manchester Short Assessment (Table 10). For analysis, we pooled all measures of quality of life together. We estimated that those in IPS conditions had a higher levels of QOL, but that the confidence intervals for the estimate included the null (Fig 6, k = 9, d = 0.30, SE = 0.19, Z = 1.60, p = 0.11, 95%CI = [-0.07 0.67], τ^2^ (SE) = 0.2955(0.1602), I^2^ = 94.90%, H^2^ = 19.59, Q(8) = 62.1751, p<0.0001). We estimated that the proportion of settings that we expect the effect size to be d>0.2 was relatively large (Prop = 0.57, 95%CI = [0.30, 0.84]) but that those that would be expected to be d<-0.2 was relatively small with a confidence interval that included zero (Prop = 0.18, 95%CI = [0, 0.40]). Length of study was not a significant moderator (Length = -0.0005, 95%CI = [-0.03, 0.03]). Given the heterogeneity and the different scales used, we also tested to see if scale was a significant moderator and we estimated that it may be with the Personal Wellbeing Index having the highest estimate (Table 11).

**Table 11:** Quality of life–scales as moderator.

	Estimate	SE	CIL	CIU
Intercept^a^	0	0.13	-0.25	0.25
MANSA	0.14	0.16	-0.17	0.46
Personal Wellbeing Index	1.90	0.26	1.39	2.42
Quality of Life Interview	0.13	0.13	-0.13	0.39
VC36-MCS	0.007	0.21	-0.40	0.41
Wisconsin Quality of Life	0.41	0.24	-0.06	0.87

a. Intercept is the Lancashire Quality of Life Profile

### Global functioning

**Fig 7:**
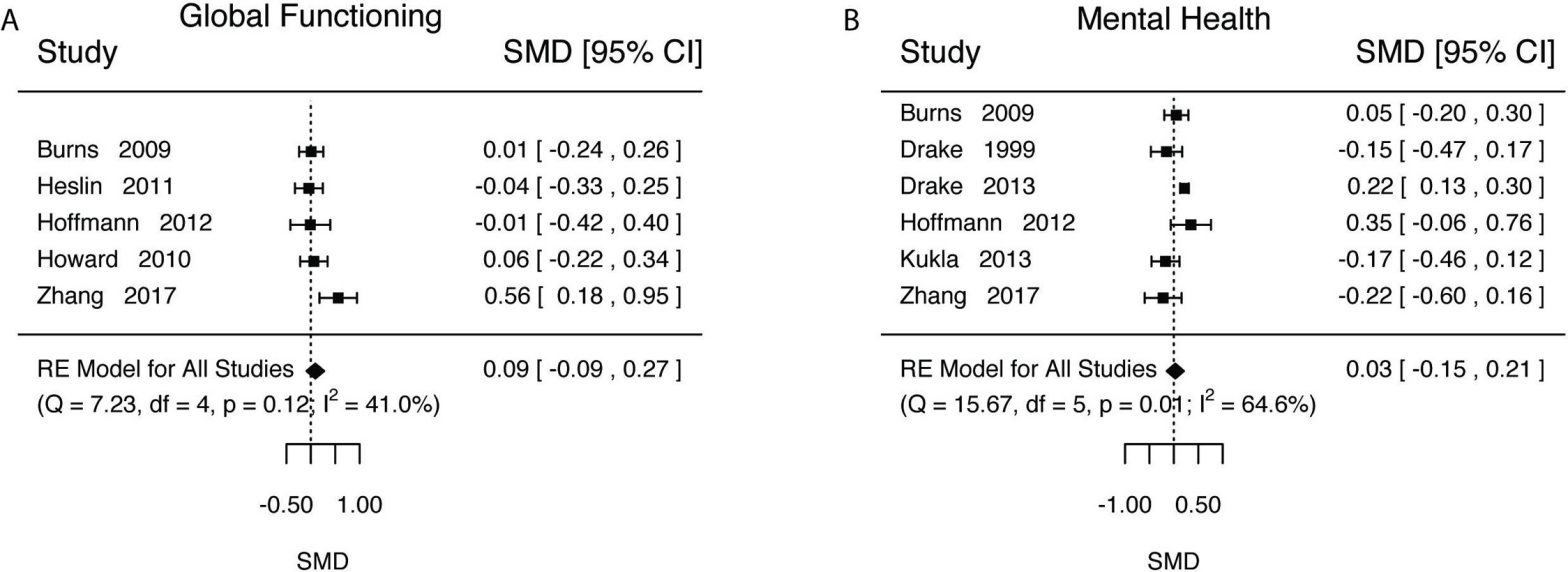
Global functioning and mental health outcomes. A) Global functioning. B) Mental health.

Several studies reported some measure of global functioning (Table 12). Global functioning measures how severe a person’s symptoms are (*i*.*e*., how big of an impact on daily life are symptoms). We estimated that those in IPS had a slightly higher global functioning, but that the confidence intervals of the estimate include the null (Fig 7A, k = 5, d = 0.09, SE = 0.09, Z = 0.9957, p = 0.3194, 95%CI = [-0.0891, 0.2731]). Our estimates of scientifically meaningful proportions were both small and the confidence intervals included the null for both directions (d>0.2, Prop = 0.21, 95%CI = [0, 76]; d<-0.2, Prop = 0.01, 95%CI = [0, 0.15]).

**Table 12:** Global functioning.

Article	IPS			TAU		
	Mean	SD	N	Mean	SD	N
Burns 2009	55.5	11.94	156	55.4	13.04	156
Heslin 2011	56.1	16.4	93	56.8	18.7	95
Hoffman 2012	55.2	9.1	46	55.3	9.1	54
Howard 2010	53.34	14.75	98	52.46	13.16	99
Zhang 2017	63.69	2.23	54	62.5	1.97	54

### General mental health

In addition to the above measures, researchers also recorded values for several other broadly mental health related measures. Here, we pooled reported outcomes on the Brief Psychiatric Rating Scale, the Positive and Negative Syndrome Scale, and the mental component of the Short-Form Health Survey (Table 13).

**Table 13:** Mental health.

Article	Scale	IPS			TAU		
		Mean	SD	N	Mean	SD	N
Burns 2009	PANSS	29.3	7.82	132	28.9	7.87	120
Drake 2013	Other	38.79	13.3	1004	35.91	13.18	1051
Drake 1999	BPRS	39.2	10.23677	74	41	13.42541	76
Hoffman 2012	PANSS	23.4	5.2	46	21.7	4.5	54
Kukla 2013	PANSS	59.9	15.97	92	62.95	19.16	95
Zhang 2017	BPRSS	23.46	1.99	54	23.87	1.71	54

We estimated a very small effect and that the confidence intervals of the estimate include the null (Fig 7B, k = 6, d = 0.03, SE = 0.09, Z = 0.3320, p = 0.7399, 95%CI-0.15, 0.21], τ^2^ (SE) = 0.03(0.03), I^2^ = 64.55%, H^2^ = 2.82, Q(5) = 15.67, p = 0.0078). Our estimates of scientifically meaningful proportions were both small and the confidence intervals included the null for both directions (d>0.2, Prop = 0.15, 95%CI = [0, 0.51]; d<-0.2, Prop = 0.08, 95%CI = [0, 0.36]). Experiment length was not a significant moderator (Length = 0.03, SE = 0.02, Z = 1.46, p = 0.14, 95%CI = [-0.01, 0.08]).

## Discussion

From the above results, it is clear that supported employment (SE) as deployed via individual placement and support (IPS) treatments is significantly better than treatment as usual (TAU) on all vocational outcomes. Furthermore, there is some evidence that IPS may also improve quality of life and maybe global functioning, but more research with larger sample sizes are needed to know for sure.

For all vocational outcomes, those in IPS treatments had higher rates of competitive employment at any point during the study (log(RR) = 0.49, 95%CI = [0.38, 0.60], Fig 2A) and at the end of the study (log(RR) = 0.58, 95%CI = [0.42, 0.74], Fig 2B), shorter time to first competitive employed job (d = -0.31, 95%CI = [-0.46, -0.16], Fig 3), longer job tenure (d = 0.56, 95%CI = [0.33, 0.79], Fig 4A), longer durations of work (d = 0.46, 95%CI = [0.35, 0.57], Fig 4B), and higher total income (d = 0.48, 95%CI = [0.36, 0.59], Fig 5).

In addition to vocational outcomes, we also examined non-vocational outcomes. All our estimates were small and included the null (quality of life d = 0.30, 95%CI = [-0.07, 0.67], Fig 6; global functioning d = 0.09, 95%CI = [-0.09, 0.27], Fig 7A; mental health d = 0.03, 95%CI = [-0.15, 0.21], Fig 7B). For quality of life, additional analysis estimating the proportion of settings did provide some additional insight pointing to possible positive effects. We estimated that the proportion of settings that would have an effect of d>0.2 was 0.57 (95%CI = [0.30, 0.84]). This latter result is suggestive that there may be a significant effect of IPS on these outcomes, but that the effect is sensitive to the setting. If this is a true effect, a possible route of causation for increased quality of life and other non-vocational outcomes could be via the competitive employment. A recent study using mediation analysis suggest improvement in quality of life may be fully mediated via competitive employment [54].

### Methodological interpretation and critique

In the research reviewed here, researchers often reported that a result was not significant, but gave no estimate of the result (*e*.*g*., means and standard deviations). Because of this, we were unable to include all outcomes that researchers said that they had measured. We urge all researchers going forward to report all estimated values, even for non-significant results based upon null hypothesis testing. Although a result may not rise to the level of statistical significance in a single study, through the use of meta-analysis future researchers might be able to tease out that there is a significant population level effect present.

We also suggest that researchers base sample sizes upon the smallest effect that they wish to detect, not the main outcome, especially if secondary outcomes are expected to have considerably smaller effect sizes than the main outcome.

In subsequent meta-analyses we would also encourage researchers to report not only the pooled effect estimate but also the estimated proportion of settings in which there are scientifically meaningful effect sizes, both positive and negative. In some cases, these proportion measures may not be especially additionally informative. For example, with achieving competitive employment, the pooled estimates and the proportion measures gave very similar information of consistent beneficial effects. But in other settings, a wide confidence interval that includes the null or a near-null pooled estimate may be principally driven by effect heterogeneity. Thus with quality of life, although the point estimate was small and its confidence intervals include the null (d = 0.30, 95% CI = [-0.07, 0.67]), the estimated proportion of studies for which the effect size was d>0.2 was substantial (Prop = 0.57, 95% CI = [0.30, 0.84]), indicating that for a number of studies there was substantial improvement. Furthermore, the estimated proportion of studies that would be in the opposite direction with d<-0.2 included was not entirely negligible but confidence interval did include the null (Prop = 0.18, 95%CI = [0, 0.40]). We believe more routine reporting of such metrics, especially in contexts like SE where effects can be quite variable, would help provide a more nuanced understanding of results.

### Can we expand supportive employment to more groups?

SE and IPS were originally developed for and used with populations that had severe mental illness. Given the effectiveness of IPS, are there additional groups that could benefit. The answer appears to be a ‘yes’ from our above results and past literature.

A number of studies have looked IPS in the US veteran population. The majority of studies looked at veterans with spinal cord injuries. Ottomanelli and colleagues show that veterans with SCI have improved CE outcomes when placed in SE/IPS treatments compared to standard ones [16].

There is also work done on PTSD and veterans, which also found that SE/IPS was better than TAU [55]. One study looked at moderating effect of PTSD on outcomes for SE [56] and found that PTSD groups were less likely to be employed in the SE and other conditions. The difference seemed to be most evident in the SE treatment group.

Previous research reported that for populations with schizophrenia or schizoaffective disorder, IPS proved more effective than TAU as measured by subjects finding competitive employment (57% vs. 29%)[44].

Although research in SE first looked at those with severe mental illness, it now includes other disabilities such as spinal cord injury [16, 24] and affective disorders [17]. There were a number of other subject populations that were not explicitly detailed in the results because they were limited to a single study per population. These include those on social security disability insurance [57], young adults [58], emerging adults ages 17–20 [59], those with general affective disorders [17], homeless populations [42], those receiving methadone treatment [60], minority groups [61], those with a criminal justice history [29, 62], and those with musculoskeletal injuries [38]. All of these studies showed that those in IPS faired better than those in the TAU control on the main measured outcome of competitive employment rates.

That there is the possibility for expanding the reach of SE via IPS raises some interesting possibilities as well as serious questions. The most obvious question is “What is the limit of IPS?” Could IPS be provided to those without any noticeable or known disability? The answer appears to be “yes” that, in the limit, IPS could be provided to everyone. But what would this look like? It would boil down to having dedicated personal to help subjects find competitive employment.

Part of the IPS treatment is that it provides a means to help those looking for work find competitive work. That this would work with other groups in general is probably not too surprising. We must remember that the initial surprise after all in this literature was that this approach could and does appear to work with those with severe mental illnesses and disabilities who had previously been thought to be ‘unfit’ for work until after their mental disabilities were resolved or at least controlled.

The utility of expanding this treatment to groups without disabilities, and whether that is at all cost-effective, is not obvious. However, expansion to other groups that have a disability or suffer from some other symptoms and who are normally encouraged to follow a two-step serial process does seem to make sense. For these groups, it would be good to understand if SE via IPS could benefit them as the current results suggest it might.

Finally, within the traditional groups that this treatment was designed to help, there is a need to better differentiate between the types of severe mental illness (SMI) that subjects generally have. SMI usually includes, but is not limited to, schizophrenia, bipolar disorders, and severe depression given that each of these may have its own etiology and neurobiological underpinnings. For example, there may be a difference in the structural (white matter) between bipolar and unipolar depression [63]. Better tracking of these groups could help to elucidate when and if IPS helps with non-vocational outcomes.

### Can we make supportive employment more effective?

As seen above, IPS is quite effective in helping certain populations gain CE. It is less certain how much better SE is than treatment as usual for other outcomes such as mental health. Our estimates suggest that there is little effect and that for some of the outcomes the observed effect is quite heterogeneous. Are there better alternatives to this implementation?

An early work by McGurk and colleagues looked at the impact of cognitive training (CT), specifically, the “Thinking Skills for Work Program”, in conjunction with SE [64]. They found that CT+SE outperformed SE alone in percentage of subjects employed. A different study looked to augment SE with cognitive training in subjects with schizophrenia [56]. They found that the added training significantly increased the outcome of subjects (30% vs. 9%). Most recently, they found that for those that didn’t respond to IPS, the individual receiving cognitive enhancement treatments (the “Thinking Skills for Work Program”) had higher employment (60% vs. 36%), worked longer (23.9 vs. 9.2 weeks), and earned more ($3,421 vs. $1,728) than the control group that had an enhanced version of SE [65].

Other researchers have looked at the effectiveness of skills training in conjunction with IPS [66]. They found that while the test group increased their knowledge of fundamental concepts of the workplace (e.g., job performance), they did not differ from controls on a host of other outcomes. The subjects (both treatment and control) had, however, already been in IPS prior to the study.

There have also been efforts to expand how IPS is done. For example, one study looked at expanding SE beyond the rigid requirements of IPS [67]. Instead of using specialized IPS delivery centers, these researchers were able to show that IPS can be effectively delivered in a ‘multi-server’ program center.

### Conclusion

There is little doubt that for the primary outcome, which was competitive employment rates, the supported employment framework and the IPS treatment is more effective than treatment as usual. There is even good evidence that the IPS treatment can be expanded and remain effective with groups other than those with severe mental illness (who were the original targets of the intervention).

Still, it must be noted that SE/IPS is not a panacea. Subjects still work below full time and still suffer from their underlying disorder. The obvious real solution would be to eliminate fully the underlying condition, but this is, of course, often difficult.

There is some evidence that even though the above is true, the cost-benefit of SE/IPS is not necessarily better than TAU. One RCT investigated the cost-benefit of SE compared to other vocational rehabilitation programs [68]. Depending on the outcome measured, SE/IPS may or may not be more effective. A fairly recent study looked at the cost-benefit of SE for veterans with spinal cord injuries and found that it was not significantly more cost-effective than TAU [69]. Also, vocational rehabilitation closure rates (i.e., success) of SE/IPS compared to TAU has been observed to be the same [70]. However, these studies do not take into account possible increases in meaning and quality of life, the “value” of which are of course difficult to quantify.

Finally, we must ask ourselves what is the ultimate purpose of SE/IPS and vocational rehabilitation and what ought the ultimate purpose truly be. If SE/IPS provides greater employment but does not help in other respects, is it worth it to have these services? SE/IPS does provide greater competitive employment and may also contribute to a higher level of quality of life. SE/IPS does not appear to significantly decrease subjects’ mental health symptoms, but neither does it increase their symptoms, though there was some evidence that the effects may be heterogeneous across studies. Examining further and understanding that heterogeneity may be essential to developing further improvements in SE interventions to enhance an even broader range of outcomes, though much work would still be required to make this a reality.

## Supporting information

S1 File PRISMA_checklist. PLoS One PRISMA Checklist.
(PDF)

S2 File Supported_employment. R script file with source code for performing analyses used in this paper.
(R) Click here for additional data file.

S3 File R data files (zipped) to use with S2 for reproducing analyses. (ZIP)
